# Obese subcutaneous adipose tissue impairs human myogenesis, particularly in old skeletal muscle, via resistin-mediated activation of NFκB

**DOI:** 10.1038/s41598-018-33840-x

**Published:** 2018-10-18

**Authors:** Mary F. O’Leary, Graham R. Wallace, Edward T. Davis, David P. Murphy, Thomas Nicholson, Andrew J. Bennett, Kostas Tsintzas, Simon W. Jones

**Affiliations:** 1 Institute of Inflammation and Ageing, MRC-ARUK Centre for Musculoskeletal Ageing Research, University of Birmingham, Birmingham, UK; 2 The Royal Orthopaedic Hospital NHS Foundation Trust, Bristol Road South, Northfield, Birmingham, B31 2AP UK; 3 Department of Clinical and Movement Neurosciences, Institute of Neurology, University College London, London, UK; 4 FRAME Alternatives Laboratory, School of Life Sciences, Faculty of Medicine & Health Sciences, University of Nottingham, Nottingham, UK; 5 MRC-ARUK Centre for Musculoskeletal Ageing Research, School of Life Sciences, Faculty of Medicine & Health Sciences, University of Nottingham, Nottingham, UK

## Abstract

Adiposity and adipokines are implicated in the loss of skeletal muscle mass with age and in several chronic disease states. The aim of this study was to determine the effects of human obese and lean subcutaneous adipose tissue secretome on myogenesis and metabolism in skeletal muscle cells derived from both young (18–30 yr) and elderly (>65 yr) individuals. Obese subcutaneous adipose tissue secretome impaired the myogenesis of old myoblasts but not young myoblasts. Resistin was prolifically secreted by obese subcutaneous adipose tissue and impaired myotube thickness and nuclear fusion by activation of the classical NFκB pathway. Depletion of resistin from obese adipose tissue secretome restored myogenesis. Inhibition of the classical NFκB pathway protected myoblasts from the detrimental effect of resistin on myogenesis. Resistin also promoted intramyocellular lipid accumulation in myotubes and altered myotube metabolism by enhancing fatty acid oxidation and increasing myotube respiration and ATP production. In conclusion, resistin derived from human obese subcutaneous adipose tissue impairs myogenesis of human skeletal muscle, particularly older muscle, and alters muscle metabolism in developing myotubes. These findings may have important implications for the maintenance of muscle mass in older people with chronic inflammatory conditions, or older people who are obese or overweight.

## Introduction

The loss of skeletal muscle mass with ageing (sarcopenia) is accompanied by an increased systemic inflammatory burden^1^ and accumulation of adipose tissue^2^, which is known to be a prolific secretor of pro-inflammatory cytokines^3^ termed adipokines. Notably, the prevalence of sarcopenia is greater in obese than in non-obese older individuals^4^ and this association has been referred to as sarcopenic obesity^5^. Sarcopenic obesity is considered to be an important public health concern in the elderly as it confers a higher risk for developing disability in the muscle functional activities of daily living, culminating in reduced quality of life^6^. Significantly, sarcopenic obesity is associated with increased levels of systemic pro-inflammatory markers after adjusting for the presence of other pro-inflammatory states such diabetes and cancer^7^.

Cross-sectional studies of the body composition of individuals across the lifespan found that total adipose tissue mass^8^ and visceral adipose tissue (VAT) mass^9^ negatively correlated with skeletal muscle mass. Indeed, the cross-sectional Health, Aging, and Body Composition (Health ABC) study of 3075 men and women aged 70–79 years demonstrated that those with high systemic concentrations of TNFα and IL-6 had a smaller mid-thigh muscle cross-sectional area and decreased grip strength^10^. Furthermore, circulatory levels of adiponectin and resistin are increased in old individuals, compared to the young, and are inversely associated with muscle strength^11^.

Collectively, these studies indicate that sarcopenia in overweight and obese individuals is in part an adipokine-driven phenomenon. Indeed, increased adipocyte-derived pro-inflammatory cytokines and lipid metabolites may contribute to decreased regenerative capacity^12^ and myogenesis^13^ of skeletal muscle. The anti-myogenic and muscle atrophic actions of some adipokines (such as TNFα and IL-6) are well studied in this respect, with data from *in vivo* and *in vitro* studies implicating their role in mediating muscle loss^14–22^. Furthermore, a recent study demonstrated that TNFα exacerbates saturated fatty acid (palmitate)-mediated lipotoxicity of murine myoblasts^23^, indicating the importance of pro-inflammatory cytokines in the context of the adipose tissue inflammatory milieu. However, the functional effects of many other obesity-associated adipokines, including resistin, on human skeletal muscle are not well characterised. Furthermore, very few studies have examined the inflammatory milieu secreted by human subcutaneous adipose tissue (SAT).

Despite the prominent attention that visceral adipose tissue (VAT) has received as a secretor of adipokines, SAT also secretes pro-inflammatory adipokines, albeit to a lesser extent^24–26^. Importantly, SAT represents a much greater proportion of total adipose tissue mass than VAT^27–29^, and therefore may be greatly underappreciated as a contributor to the systemic inflammatory burden. To our knowledge, a single study has directly examined the effect of an inflammatory milieu secreted by human SAT adipocytes on primary human myotube morphology^24^. This particular study showed that conditioned medium from SAT adipocytes derived from lean individuals does not alter the Myotube Thickness (MTT) or Nuclear Fusion Index (NFI) of myotubes in myogenic cultures isolated from a neonate^24^. Interestingly, adipocytes isolated from obese SAT showed an intermediary inflammatory profile and negative effect on MTT between lean SAT and obese VAT. In the same study, conditioned medium from obese VAT adipocytes significantly diminished MTT but not NFI when compared with control and lean SAT. Furthermore, direct co-culture of obese VAT adipocytes with myoblasts derived from a neonate, resulted in significant reduction in the expression of the myogenic transcription factors MyoD1 and myogenin. However, the study used adipocytes, rather than whole adipose tissue to generate conditioned medium representing the secretome, and thus did not characterise the effects of adipose tissue’s complete inflammatory milieu on myogenic cultures. Critically, the stromal vascular fraction of adipose tissue, which includes preadipocytes and macrophages, is a more prolific secretor of pro-inflammatory cytokines than mature adipocytes^25^. Furthermore, the myotubes used were neonate not from adult donors. Consequently, adipokine secretion by human adipose tissue – not just that by adipocytes – must be characterised and its effect on human myofibre size and function determined.

The aim of this study was therefore to determine the effects of lean and obese SAT conditioned media secretome, and in particular the adipokine resistin, concentrations of which are known to be increased in the serum of obese individuals^30,31^, on human skeletal muscle myogenesis and metabolism using muscle cell cultures derived from both old and young individuals.

## Materials and Methods

### Skeletal muscle biopsy and myogenic culture isolation

Three young healthy subjects (2 males and 1 female; age 24.4 ± 1.5 yr; BMI 22.6 ± 2.2 kg/m^2^) and four elderly healthy individuals (2 males and 2 females; age 70.5 ± 2.8; BMI 21.8 ± 1.3 kg/m^2^) were recruited and gave written informed consent. All participants were physically active (at least 150 minutes of self-reported moderate intensity activity per week). Participants were free from cardiovascular, metabolic, neuromuscular or other diseases that might affect muscle growth and metabolism during screening. The study was approved by the University of Nottingham Medical School Ethics Committee (G11092014SoLS) and was conducted in accordance with the guidelines of the Declaration of Helsinki. A vastus lateralis muscle biopsy was obtained from each subject and the satellite cell population extracted as previously described^32^. Our isolation technique has consistently generated cultures in our laboratories that produce desmin positive multinucleated myotubes that are negative for the fibroblast marker TE7^32^. Additionally, commercially available primary human myoblasts (Thermo Fisher cat. No. A12555), isolated from a female aged 21 yr were utilised for mechanistic studies, which were cultured in the same media and conditions as the cultures that we isolated in-house.

### Generation of adipose conditioned medium secretome

Following ethical approval (UK National Research Ethics Committee 16/SS/0172), SAT was obtained intraoperatively from n = 13 lean (BMI < 25, age 68.1 ± 3.3 years) and n = 22 non-lean (BMI > 25, age 69.5 ± 1.8 years) older individuals undergoing elective total joint replacement surgery at either the Royal Orthopaedic Hospital (Birmingham, UK) or Russell’s Hall Hospital (Dudley,UK). SAT was incubated in myotube differentiation medium at a ratio of 1 g tissue to 10 mL medium for 24 h at 37 °C, 21% O_2_ and 5% CO_2_. Larger samples were divided into segments of ~1 g to ensure that the surface area of adipose tissue exposed to medium remained approximately constant. At 24 h the adipose conditioned medium (ACM) was removed, aliquoted into 5 mL sample containers and stored at −80 °C. For experimental use, the ACM was diluted 1:2 with differentiation medium, to ensure a sufficient nutrient composition to sustain myogenic differentiation.

### Immunofluorescence staining

Myotubes were differentiated for 8 d in the presence of ACM secretome or recombinant resistin protein. The details of cytokine concentrations, as well as the timing and duration of such stimulations, are described in the relevant results section and Figure legend. Media were renewed every 2 d. The culture medium was removed and the cells fixed with 2% formaldehyde in PBS for 30 min. Following permeabilization in 100% methanol for 10 min, wells were blocked with 5% goat serum in PBS for 30 min. The primary antibody was diluted (Desmin, 1:1000, Dako) in 1% BSA/PBS and 150 μL was added per well for 1 hour. Wells were subsequently incubated with 150 μL/well secondary antibody (Goat anti-Mouse IgG (H + L), Alexa Fluor® 488 conjugated, Thermo Fisher) for 1 h in the dark. Each well was washed with PBS and 150 μL/well DAPI/PBS (1:5000, Cell Signalling Technology) was added for 5 min in the dark. Wells were further washed with PBS, a drop of mountant added to each well (ProLong Diamond Antifade, Thermo Fisher) and a coverslip applied.

### Quantification of myotube thickness and nuclear fusion index

24-well plates of immunuofluoresence (IF) stained myotubes were imaged on an epifluorescence/brightfield microscope (Leica DMI6000). Triplicate wells were stimulated for each biological replicate and for each treatment condition. Multiple images were taken in each well for the quantification of myotube thickness (MTT) and nuclear fusion index (NFI). For quantification of MTT, 15 images per well were obtained using a 63x objective, the first image being obtained at a fixed starting point and subsequent images selected by moving to the next field of view in a predefined pattern. For assessment of NFI, 5 images per well were obtained in the same fashion, using a 20x objective. Image analysis was carried out by a blinded researcher, using Image J software. A myotube was defined as a desmin positive structure, containing 3 or more nuclei. The MTT of each myotube was calculated by taking the average of 5 measurements obtained along its length. The NFI was defined as the number of nuclei clearly incorporated into myotubes expressed as a proportion of the total visible nuclei in each field of view.

### Immunoblotting

Protein extraction, SDS-PAGE and immunoblotting were performed as previously described^32^. Primary antibodies for NFκB p65 (Cell Signalling Technology #6956), phosphorylated (Ser^536^) NFκB p65 (Cell Signalling Technology #3033) and resistin (polyclonal rabbit IgG, Thermo Fisher PA1-1049) were used. Anti-mouse (NA931V, GE Healthcare) and anti-rabbit (NA934, GE Healthcare) HRP-linked secondary antibodies were diluted 1:5000 in TBS-T, and blots were developed using ECL-plus (GE Healthcare, Amersham Biosciences, Amersham, UK) according to the manufacturer’s instructions. Bands were visualised on the ChemiDoc MP imaging system (Bio-Rad, UK).

### Immunoprecipitation of resistin from adipose conditioned media

70 μL Protein A Sepharose® beads (Abcam, ab193256) were incubated with 1 μg anti-resistin primary antibody (polyclonal rabbit IgG, Thermo Fisher PA1-1049) or 1 μg rabbit IgG isotype control (Sigma Aldrich, 12–370). The antibody-bead mixture was incubated for 4 h at 4 °C on a shaker. The beads were centrifuged at 3,000 g for 2 min at 4 °C and the supernatant was discarded. Beads were then washed twice with PBS, 5 mL ACM added to each bead-antibody conjugate and the ACM-bead-antibody mixture incubated for 24 h at 4 °C with rotary agitation. The mixture was centrifuged at 3,000 g for 2 min at 4 °C and the supernatant (ACM) was retained and stored at −80 °C. The antibody-bead conjugates were washed in PBS as before. The antigen-antibody complexes were eluted from the sepharose beads by the addition of 50 μL 2x Laemmli sample loading buffer. The elutes were incubated at 50 °C for 10 min and stored at −80 °C in advance of their use in immunoblotting for the detection of resistin.

### Multiplex immunoassay

Cytokine and chemokine concentrations were quantified in ACM secretome by multiplex magnetic bead-based immunoassay (Luminex® Screening Assay, R&D Systems) according to the manufacturer’s instructions. 50 µL of a 1x antibody magnetic bead stock (Adiponectin, Serpin E1, Aggrecan, Amphiregulin, CCL11, CCL2, CCL3, CCL20, Chemerin, CXCL10, Dkk1, Galectin-1, gp120, IL-1β, IL-10, IL-15, IL-7, visfatin, TNFα, Galectin-3BP, Lipocalin-2, CCL4, FABP4, LIF, Leptin, IL-6, Resistin) was added to each well of a flat bottom black plate. 50 µL of undiluted sample or standard were added in duplicate to the plate. The plate was then sealed and incubated for 2 h on an orbital rotator. The plate was washed three times with a magnetic plate washer (Bio-Plex Pro™ Wash Station, Bio-Rad) using the wash buffer provided. 50 µL of a biotinylated antibody cocktail was added to each well; the plate was resealed and incubated for 1 h on the orbital rotator. The wash steps were repeated as before and 50 µL of the provided streptavidin-PE was added to the wells. The plate was incubated on the orbital rotator for 30 min and the wash steps repeated for a final time. Finally, the beads were resuspended in 200 µL wash buffer and the analytes were quantified by the Luminex® 200 multiplex analyser (Luminex® Corporation).

### Oil Red O staining for assessment of intramyocellular lipid content (IMCL)

Oil Red O staining was carried out on 24-well plates of myotubes that had been previously IF stained using a protocol adapted from Koopman *et al*.^33^. In brief, the wells were first washed 3 times with PBS. An Oil Red O stock solution (0.5% (w/v) Oil red O in 60% triethyl-phosphate in ddH_2_O) was diluted 3:2 in double distilled (dd)H_2_O, and filtered through a 0.22 µM filter. 150 μL Oil Red O working solution was added to each well for 1 h. The wells were washed as before in PBS, a drop of mountant (ProLong Diamond Antifade, Thermo Fisher) added to each well and a coverslip applied. Brightfield imaging was carried out on a microscope (Leica DMI6000). The Oil Red O positive area was quantified by Image J analysis. Briefly, each image was converted to a grayscale 8-bit format and the threshold was adjusted to highlight pixels of intensity between 90–110. The total Oil Red O area of this binary image was calculated by Image J.

### Seahorse XFe96 analysis of myotube metabolic function

A Seahorse XFe96 analyser (Agilent Technologies, Santa Clara, California, United States) was used to conduct a Seahorse XF Mito Stress Test in accordance with the manufacturer’s recommendations. Myoblasts (2.5 × 10^4^ cells per well) were seeded to 0.2% gelatin-coated Seahorse XFe96 Cell Culture Microplates. At 24 h the cells were differentiated. Treatment conditions were assayed in quadruplicate and the assay was run at differentiation day 8. The XF Cell Mito Stress Test was also modified by the addition of Seahorse XF Palmitate-BSA FAO Substrate to allow the measurement of endogenous and exogenous fatty acid oxidation; this procedure was carried out according to the manufacturer’s instructions. Relative mitochondria content was determined by staining myotubes for 24 h with 100 nM Mitotracker (ThermoFisher, UK).

### Statistical analysis

Data analysis was carried out using IBM SPSS Statistics 21. All data are presented as means ± SEM of biological replicates. The normality of data was established by a Shapiro-Wilk test, whereas Levene’s test was used to establish equality of variances. For parametric data involving two treatment conditions unpaired t tests were used. Non-parametric data were analysed by Mann-Whitney U tests. Where data involving more than two treatment conditions were normally distributed, comparison was performed by a one-way or two-way analysis of variance (ANOVA) with post-hoc Bonferroni correction. Where such data were non-parametric, differences between conditions were analysed by Mann–Whitney U test with post-hoc Holm’s sequential Bonferroni correction. A p value of <0.05 was considered statistically significant. Details of the statistical tests used for each data set can be found in the relevant figure legend.

## Results

### Quantification of adipokine in the secretome of subcutaneous adipose tissue from lean and non-lean individuals

We initially profiled the concentrations of 22 adipokines in SAT conditioned media (ACM) collected from a cohort of normal weight (BMI < 25, n = 13) and overweight/obese (BMI > 25, n = 22) older individuals by multiplex magnetic bead-based immunoassays (Table 1).

**Table 1:** The Inflammatory Secretory Profile of Normal weight **(**Lean**)** and overweight/obese **(**non-Lean**)** Subcutaneous Adipose Conditioned Medium.

	Lean (BMI < 25) (Mean ± SEM, pg/mL)	Non-Lean (BMI > 25) (Mean ± SEM, pg/mL)	P-value (Lean vs. non-lean)
Adiponectin	28716 ± 4524	28841 ± 3779	0.18
Aggrecan	578, 559–606	530, 468–567	0.07^¶^
Amphiregulin	532 ± 137	703 ± 67	0.22
Chemerin-1	2655 ± 673	3450 ± 583	0.44
Eotaxin	95 ± 41	61 ± 12	0.81
FABP4	38 × 10^4^, 27 × 10^4^–81 × 10^4^	27 × 10^4^, 23 × 10^4^–39 × 10^4^	0.06^¶^
Galectin-1	5.3 × 10^4^ ± 0.6 × 10^4^	5.2 × 10^4^ ± 0.3 × 10^4^	0.85
GP130	29001 ± 10480	31824 ± 7342	0.83
IL-10	2.25 ± 0.56	2.59 ± 0.39	0.62
IL-15	1.81 ± 0.52	2.32 ± 0.37	0.45
IL-1β	12.02 ± 1.59	11.94 ± 1	0.96
IL-6	507, 436–1044	1528, 719–2889	0.12^¶^
IL-7	3.02 ± 0.27	3.04 ± 0.29	0.96
Leptin	11335 ± 2592	12210 ± 2467	0.83
MCP-1	2372 ± 924	1540 ± 406	0.34
MIP1a	363 ± 54	303 ± 32	0.33
MIP1b	101 ± 37	125 ± 24	0.58
MIP3a	85 ± 25	164 ± 51	0.97
Resistin	1207 ± 225	1778 ± 109	0.01
Serpin E1	4156, 1337–6761	10565, 3420–13450	0.02^¶^
TNFα	10.43 ± 1.57	10.43 ± 1.17	0.99
Visfatin	114, 1007–1827	917, 2051–2417	0.91^¶^

Adipokine concentrations were determined by multiplex magnetic bead-based cytokine assays in ACM from lean (BMI < 25, n = 13) and non-lean (BMI > 25, n = 22) subjects. Data are presented as mean ± SEM where normally distributed and as median, 25^th^ percentile-75^th^ percentile where not normally distributed (marked^¶^). FABP4 = Fatty acid binding protein 4, GP130 = glycoprotein 130, MCP-1 = Monocyte chemoattractant protein 1, MIP1a = Macrophage inflammatory protein 1a, MIP1b = Macrophage inflammatory protein 1b, MIP3a = Macrophage inflammatory protein 3a. IL = Interleukin; TNFα = Tumor Necrosis Factor alpha.

Comparing the ACM from the 2 groups, there was no significant difference in the concentration of the prominent adipokines leptin and adiponectin, the concentration of well-known pro-inflammatory cytokines IL-1β and IL-6, or in the concentration of the anti-inflammatory cytokine IL-10. However, the mean concentration of resistin was significantly greater (p < 0.05) in the ACM from overweight/obese older individuals (1778 ± 109 pg/mL) compared to the ACM collected from normal weight older individuals (1207 ± 225 pg/mL). Furthermore, the median concentration of serpin E1 was significantly greater (p < 0.05) in the ACM from overweight/obese (median = 10565, IQR = 3420–13450 pg/mL) compared to the ACM from normal weight older individuals (median = 4156, IQR = 1337–6761 pg/mL). Of note, there was also a trend for the median concentration of fatty acid binding protein 4 (FABP4) to be lower in overweight/obese ACM compared to the normal weight, although this did not reach significance (Table 1).

### Obese subcutaneous adipose tissue secretome impairs human myogenesis of old, but not young, muscle cells

**Figure 1:**
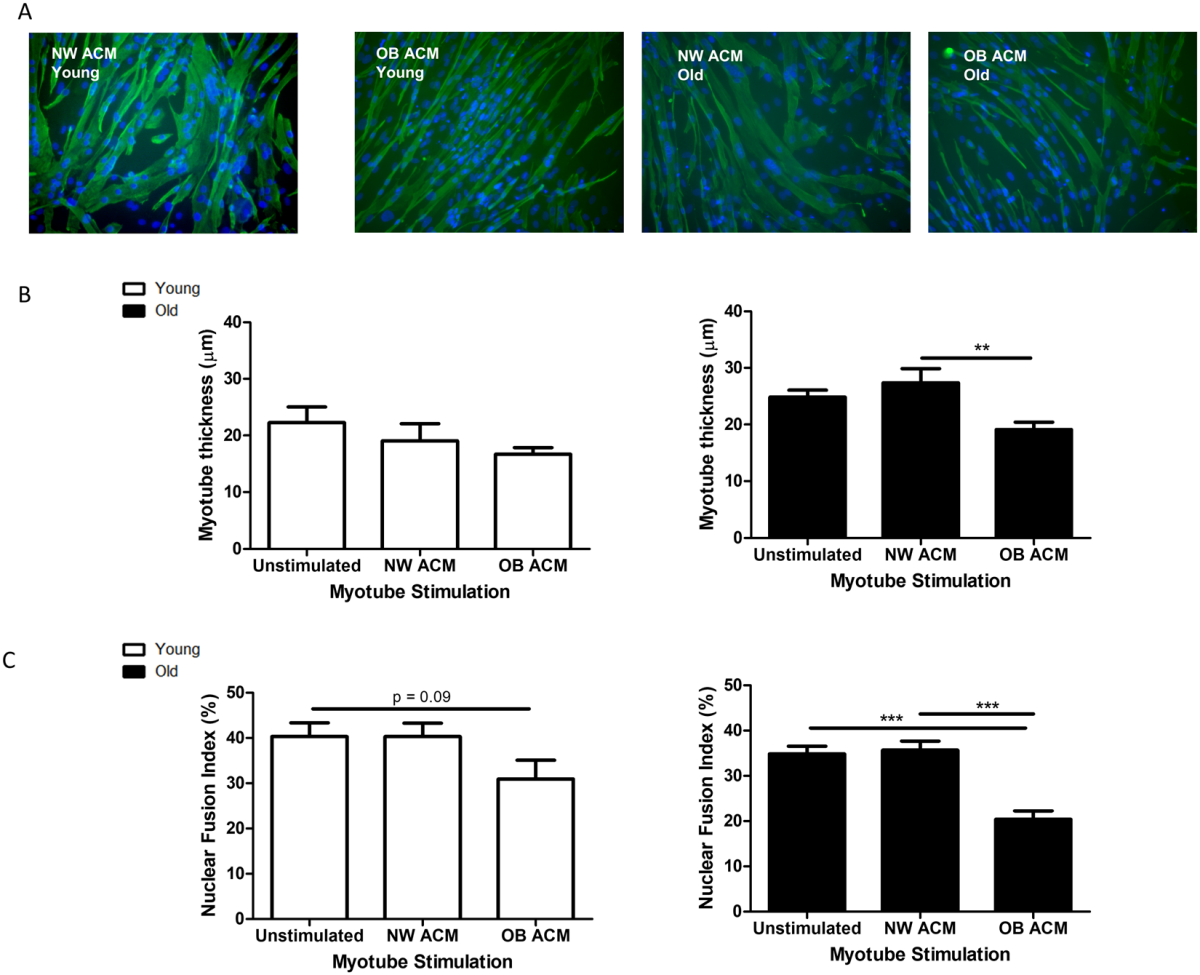
Obese subcutaneous adipose conditioned medium inhibits myotube formation in differentiating human myoblasts. Subconfluent myoblasts were switched to unconditioned differentiation medium or differentiation medium that had previously been conditioned with adipose tissue from normal weight (NW ACM, n = 3; BMI < 25 kg/m^2^) or obese individuals (OB ACM, n = 3; BMI > 30 kg/m^2^). Each young (18–30 yr) biological replicate was paired with one from the old (>65 yr) experimental group, with both being stimulated with the same ACM samples. Media were renewed every 2 d. At 8 d, myotubes were fixed, immunofluorescence stained for desmin and with DAPI and imaged on an epifluorescence microscope. (**A**) Representative images at 20x magnification. (**B**) Myotube thickness data represent the mean ± SEM of n = 3 biological replicates. Each biological replicate comprises 150 total measurements taken at 63x magnification from 30 myotubes per treatment condition. (**C**) Nuclear fusion index data are expressed as mean ± SEM values of n = 3 biological replicates. Each biological replicate comprises 15 images taken at 20x magnification. **p < 0.01, ***p < 0.001 by Mann-Whitney U test with post-hoc Holm’s sequential Bonferroni adjustment.

Having determined the concentrations of adipokines secreted from lean and non-lean SAT, we then sought to determine the effect of the ACM secretome derived from the SAT of normal weight (NW, BMI < 25) and obese (OB, BMI > 30) individuals on myotube formation. Subconfluent myoblasts from young and old lean, healthy subjects (n = 3 per group) were switched to unconditioned differentiation medium, NW ACM or OB ACM. Each young biological replicate was stimulated together with one from the old experimental group such that both young and old replicates were stimulated with the same NW and OB ACM sample. Media were renewed every 2 d. At 8 d, myotubes were fixed, IF stained for desmin and DAPI and imaged on an epifluorescence microscope (Fig. 1A).

Myotubes from elderly subjects that were stimulated with OB ACM (Fig. 1B) were 30% thinner (19.1 ± 1.3 µm) compared to their NW ACM counterparts (27.4 ± 2.5 µm, p < 0.01). The relative changes in myotube thickness (MTT) were mirrored by measurements of myotube area (Supplementary Fig. 1). The NFI of elderly myogenic cultures (Fig. 1C) was also significantly diminished by OB ACM (20.4 ± 1.9%) compared to NW ACM (35.7 ± 2.0%,p < 0.001). Young myotubes were not significantly affected by stimulation with the same ACM samples, although a trend (p = 0.09) of reduced NFI was observed when incubated with OB ACM (Fig. 1B,C).

### The adipokine resistin impairs human myogenesis

**Figure 2:**
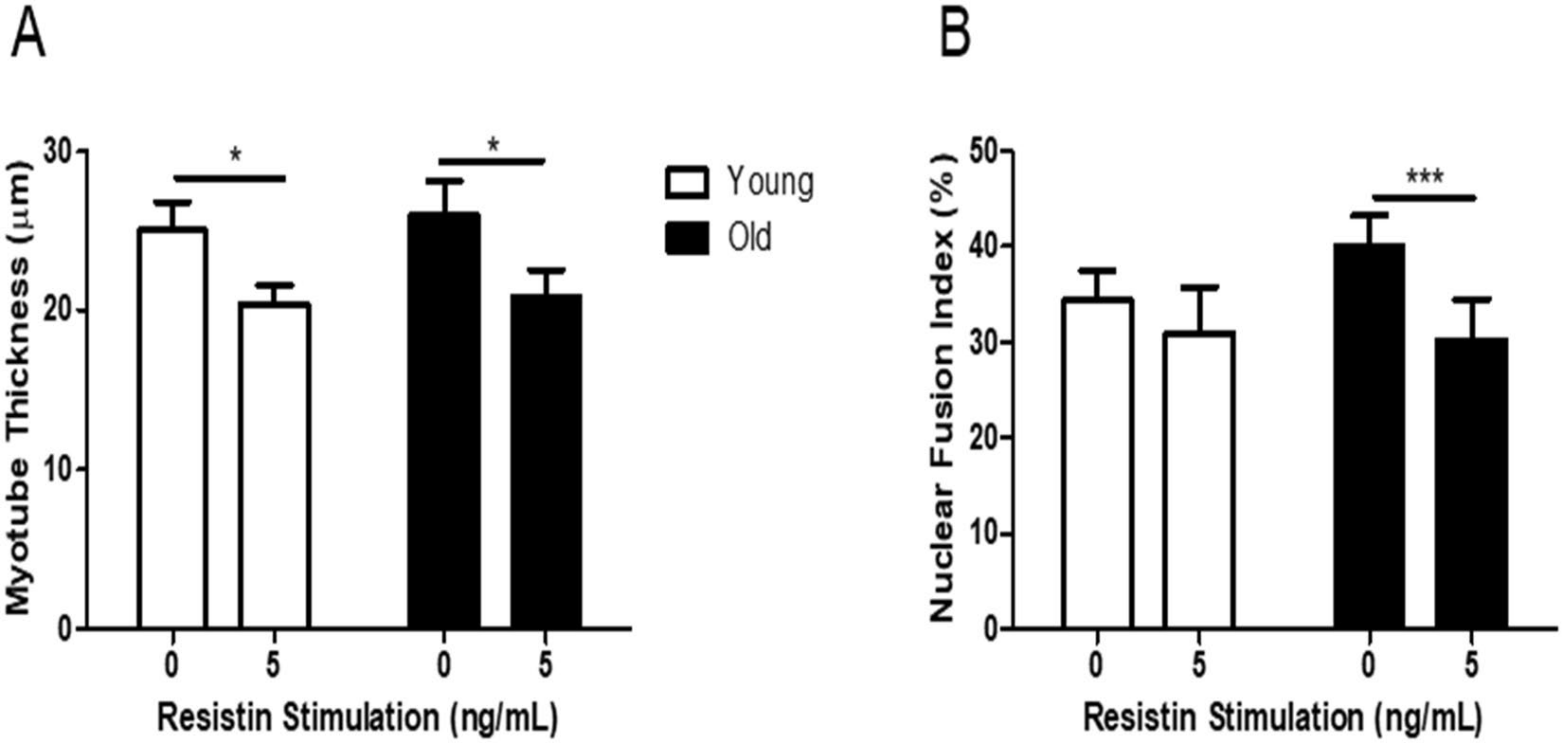
Recombinant resistin impairs myotube formation in myotubes derived from young and elderly subjects. Subconfluent myoblasts from young and elderly subjects were switched to differentiation media (with or without 5 ng/mL recombinant resistin). Media were renewed every 2 d. At 8 d, myotubes were fixed, immunofluorescence stained for desmin and with DAPI and imaged on an epifluorescence microscope. (**A**) Myotube thickness data represent the mean ± SEM of n = 3 biological replicates. Each biological replicate comprises 150 total measurements taken at 63x magnification from 30 myotubes per treatment condition. (**B**) Nuclear fusion index data are expressed as mean ± SEM values of n = 3 biological replicates. Each biological replicate comprises 15 images taken at 20x magnification. *p < 0.05, ***p < 0.001 by unpaired t test.

Since the adipokine resistin was significantly elevated in the ACM of overweight/obese individuals, we next examined the effect of stimulating myoblasts with recombinant resistin during their differentiation to myotubes. Subconfluent myoblasts from young (n = 3) and elderly (n = 3) subjects were switched to differentiation media or differentiation media containing recombinant resistin (5 ng/mL). The concentration of 5 ng/mL resistin was chosen since it represented the upper end of those concentrations observed in OB ACM. Media were renewed every 2 d. At 8 d, cultures were fixed, IF stained for desmin and with DAPI, imaged on an epifluorescence microscope and MTT and NFI were quantified as previously described. Resistin significantly reduced MTT in both young (25.0 ± 1.8 µm unstimulated control vs 20.4 ± 1.2 µm resistin stimulated, p < 0.05) and old (25.9 ± 2.2 µm unstimulated control vs 20.8 ± 1.7 µm resistin stimulated, p < 0.05) myogenic cultures (Fig. 2A). Resistin significantly diminished NFI in old cultures only (40.1 ± 3.2% unstimulated control vs 30.2 ± 4.3% resistin stimulated, p < 0.001) (Fig. 2B).

### Depletion of resistin from obese subcutaneous adipose tissue secretome improves myogenesis

**Figure 3:**
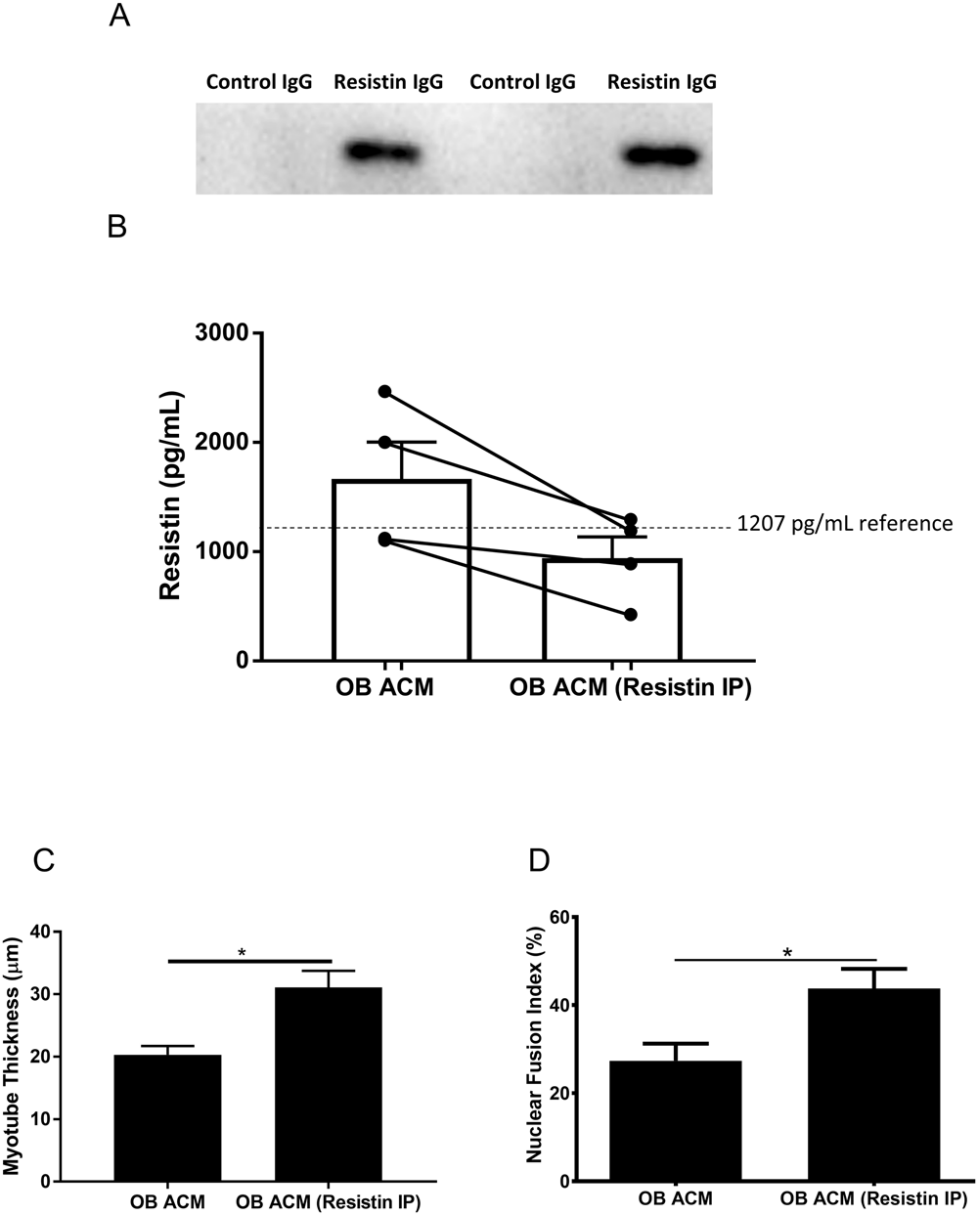
Immunoprecipitation of resistin from obese subcutaneous adipose conditioned medium secretome (OB ACM) improves myogenesis. Resistin was immunoprecipitated from OB ACM using resistin antibody-agarose bead conjugates (OB ACM – resistin IP). IgG isotype antibody control-agarose bead conjugates were used on the same samples as a control (OB ACM). (**A**) Resistin protein is detected by immunoblotting of resistin-antibody lysates but not IgG control lysates following immunopreciptation. Full sized western blot included in Supplementary Fig. 6. (**B**) Depletion of resistin in OB ACM following resistin immunoprecipitation as determined by ELISA. The dotted horizontal line illustrates the reference value of 1207 pg/mL for the concentration of resistin in NW ACM. (**C**) Subconfluent, commercially available primary human skeletal myoblasts from a female aged 21 yr were switched to either OB ACM differentiation media (OB ACM, n = 4) or to resistin-depleted OB ACM differentiation media (OB ACM Resistin IP, n = 4). Media were renewed every 2 d. At 8 d, myotubes were fixed, immunofluorescence stained for desmin and with DAPI and imaged on an epifluorescence microscope. Nuclear fusion index data are expressed as mean ± SEM values of n = 3 independent experiments. Each independent experiment comprises 15 images taken at 20x magnification. (**D**) Myotube thickness data represent the mean ± SEM of n = 3 independent experiments. Each independent experiment comprises 150 total measurements taken at 63x magnification from 30 myotubes per treatment condition. *p < 0.05 vs OB ACM by unpaired t test.

The effect of resistin on myogenesis was then validated by depletion of resistin from OB ACM secretome by immunoprecipitation. Firstly, in order to confirm the success of the immunoprecipitation, antibody conjugates were lysed and analysed by Western blotting for the detection of bound resistin (Fig. 3A). Resistin was detected in the resistin antibody conjugates lysates but not in the IgG control conjugate lysates (Fig. 3A). Secondly, the OB ACM was analysed before and after resistin immunoprecipitation for the concentration of resistin by ELISA, employing a different anti-resistin antibody than the immunoprecipitation procedure. Resistin concentrations in OB ACM were diminished following resistin immunoprecipitation, to a level similar to that found in NW ACM (Fig. 3B).

To examine the effect of resistin-depleted OB ACM secretome on myogenesis we utilised commercially available primary human myoblasts, which we first validated as responding in a similar way to our in-house cultures (Supplementary Fig. 2). Myoblasts were then switched to differentiation media containing either normal OB ACM, or resistin-depleted OB ACM. Media was renewed every 2 days as previously performed and myotubes fixed and stained at 8 days for the quantification of MTT and NFI. Myoblasts cultured with the resistin-depleted OB ACM exhibited significantly increased MTT (30.8 ± 2.8 µm vs 20.1 ± 1.3 µm, p < 0.05) and NFI (43.8 ± 4.5% vs 27.4 ± 3.9%, p < 0.05), compared to normal OB ACM (Fig. 3C,D).

### Resistin inhibits myogenesis by activation of the classical NFκB pathway

Classical NFκB pathway signalling is a negative regulator of myogenesis^34,35^. Furthermore, in multiple cell types, resistin has been shown to activate NFκB signalling^36–38^. Therefore, we next investigated whether the resistin-mediated effects on myogenesis were *via* NFκB activation.

**Figure 4:**
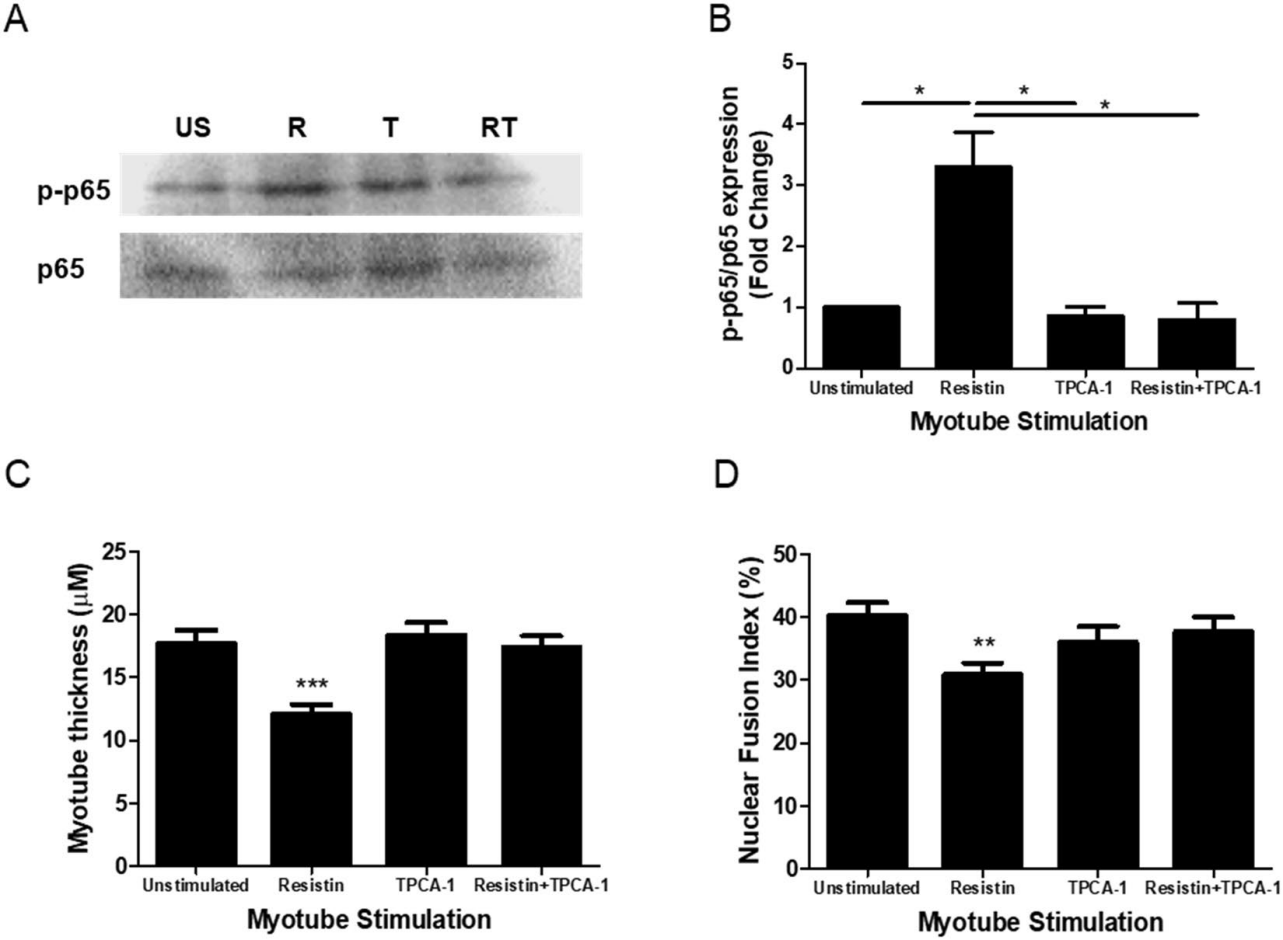
Resistin exerts its anti-myogenic effects via activation of the classical NFκB pathway. (**A**,**B**) Subconfluent primary human skeletal myoblasts from a female aged 21 yr were switched to differentiation media (with or without 5 ng/mL recombinant resistin ±40 nM TPCA-1) for 48 h. Phospho-p65 (Ser536) and total p65 were detected by immunoblotting. US = unstimulated, R = resistin, T = TPCA-1, RT = resistin + TPCA-1. Data are expressed as mean ± SEM values of n = 4 independent experiments. *p < 0.05 by one-way ANOVA with post-hoc Bonferroni correction. Full sized western blot included in Supplementary Fig. 7. (**C**,**D**) Subconfluent primary human skeletal myoblasts from a female aged 21 yr were switched to differentiation media (with or without 5 ng/mL recombinant resistin ± 40 nM TPCA-1). Media were renewed every 2 d. At 8 d, myotubes were fixed, immunofluorescence stained for desmin and with DAPI and imaged on an epifluorescence microscope. Myotube thickness data represents the mean ± SEM of n = 3 independent experiments. Each independent experiment comprises 150 total measurements taken at 63x magnification from 30 myotubes per treatment condition. Nuclear fusion index data are expressed as mean ± SEM values of n = 3 independent experiments. Each independent experiment comprises 15 images taken at 20x magnification. **p < 0.01, ***p < 0.001 vs unstimulated control by Mann-Whitney U test with post-hoc Holm’s sequential Bonferroni adjustment.

Myogenic cultures differentiated for 48 h in the presence of OB ACM induced the phosphorylation (serine^536^) of p65 (p-p65), which was inhibited by the IKKβ inhibitor 5-(p-Fluorophenyl)-2-ureido]thiophene-3-carboxamide (TPCA-1; Supplementary Fig. 3). Similarly, myogenic cultures differentiated for 48 h in the presence of 5 ng/mL recombinant resistin also displayed a significant increase in p65 phosphorylation; such phosphorylation was inhibited by the addition of TPCA-1 (Fig. 4A,B). Having established that resistin activates the classical NFκB signalling pathway during myogenesis, the ability of TPCA-1 to rescue myotubes from the anti-myogenic actions of resistin was explored. As before, 8 d resistin stimulation of differentiating myogenic cultures significantly diminished MTT and NFI, a phenomenon that was completely reversed by co-incubation with TPCA-1 (Fig. 4C,D).

### Resistin is a metabolic stressor of developing myotubes

**Figure 5:**
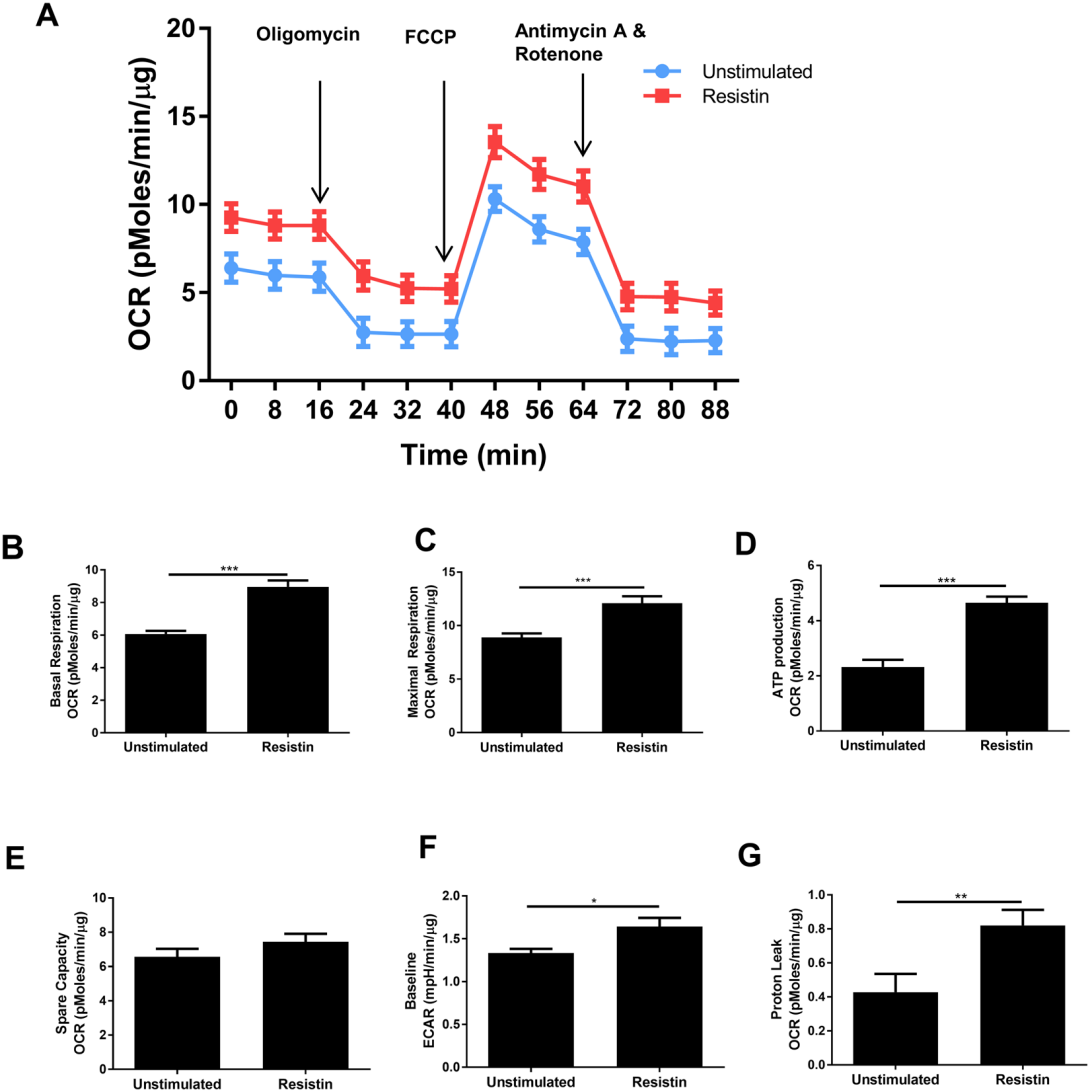
Resistin is a metabolic stressor of developing human myotubes. Subconfluent primary human skeletal myoblasts from a female aged 21 yr were switched differentiation media (with or without 5 ng/mL recombinant resistin). Media were renewed every 2 d. At 8 d, an XF Mito Stress Test was carried out on the Seahorse XFe96 analyser. (**A**) Shows the timecourse profile of Oxygen Consumption Rate (OCR) during a Mito stress test (where oliogomycin, FCCP, Antimycin A and rotenone are added sequentially) in unstimulated and resistin stimulated myoblasts. (**B**–**G**) Show the calculated mean values of basal respiration, maximal respiration, ATP production, spare capacity, baseline ECAR and proton leak in unstimulated and resistin stimulated myoblasts. Data are expressed as mean ± SEM values of n = 4 independent experiments. Each independent experiment comprises data from quadruplicate measurements. *p < 0.05, **p < 0.01 ***p < 0.001 by Mann Whitney U test.

Myogenic differentiation is associated with mitochondrial biogenesis and an increased reliance on oxidative phosphorylation^34,39^. Furthermore, alterations in myogenic differentiation capacity and mitochondrial function often coexist^40–42^. Therefore, a Seahorse® XF Mito Stress Test was conducted on myotubes that had been differentiated for 8 d with or without the presence of 5 ng/mL recombinant resistin (Fig. 5A). Resistin increased basal respiration (9.0 ± 0.4 vs 6.1 ± 0.2 pmoles/min/µg, p < 0.0001), maximal respiration (12.1 ± 0.7 vs 8.9 ± 0.4 pmoles/min/µg, p < 0.001), ATP production (4.6 ± 0.2 vs 2.3 ± 0.3 pmoles/min/µg, p < 0.0001), extracellular acidification rate (ECAR; 1.64 ± 0.10 vs 1.33 ± 0.05 mpH/min/µg, p < 0.05) and proton leak (0.82 ± 0.09 vs 0.43 ± 0.11 pmoles/min/µg, p < 0.01), compared to unstimulated control (Fig. 5B–G). However, we observed no significant difference in mitochondrial number between unstimulated and resistin stimulated myotubes, as determined using Mitotracker (Supplementary Fig. 4).

**Figure 6:**
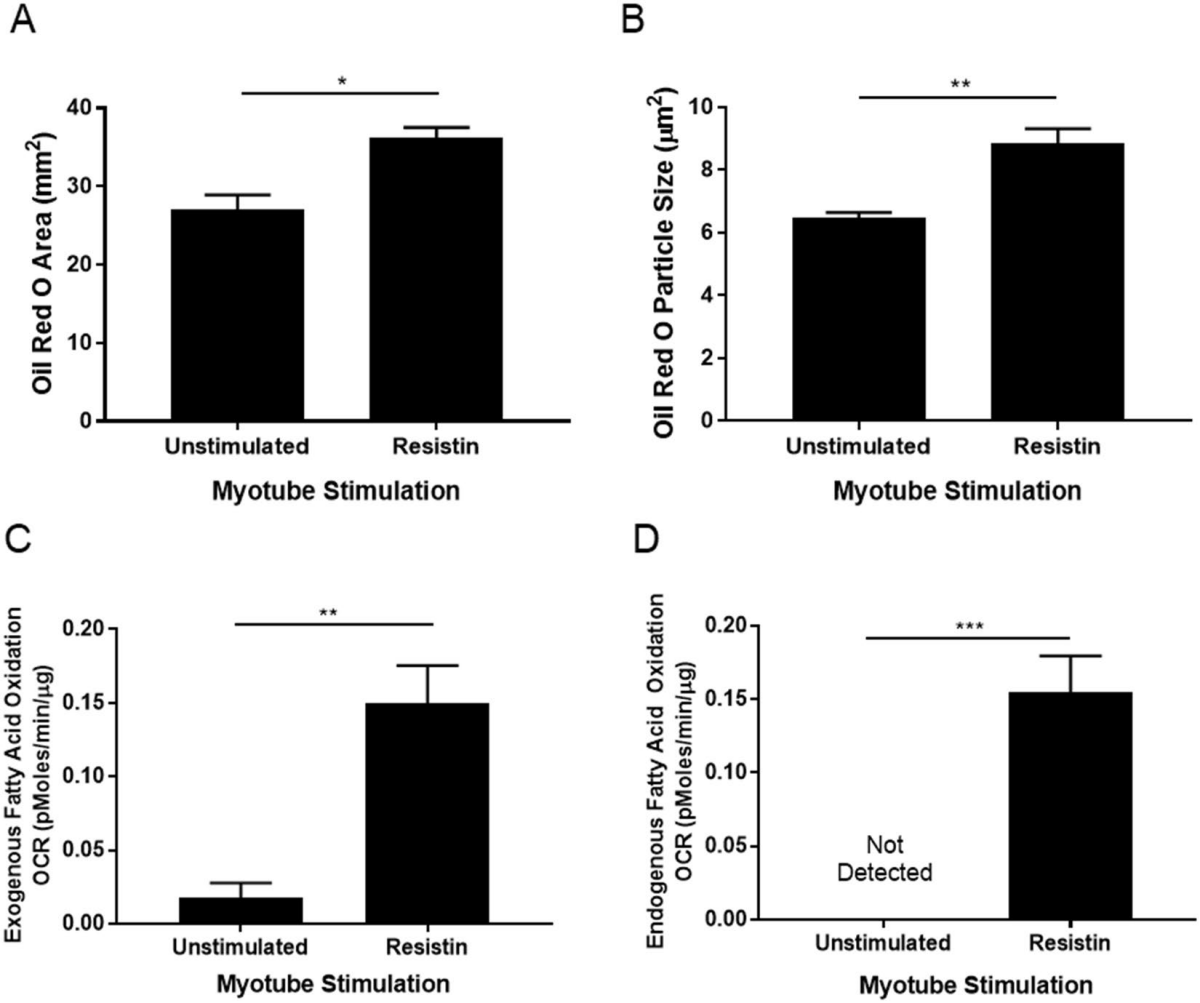
Recombinant resistin stimulation of developing myotubes from commercially available primary human skeletal myoblasts causes an accumulation of IMCL. Subconfluent primary human skeletal myoblasts from a female aged 21 yr were switched differentiation media (with or without 5 ng/mL recombinant resistin). Media were renewed every 2 d. (**A**,**B**) At 8 d, myotubes were fixed, permeabilised in 0.2% Triton X and stained with Oil Red O. Myotubes were imaged on a brightfield microscope and the Oil Red O positive area was quantified using Image J software. Data are expressed as mean ± SEM values of n = 3 independent experiments. Each independent experiment replicate comprises data from 15 images taken at 20x magnification. *p < 0.05, **p < 0.01 by t test. (**C**,**D**) At 8 d an XF palmitate-BSA FAO mitochondrial stress test was carried out on the Seahorse XFe96 analyser. Data are expressed as mean ± SEM values of quadruplicate measurements. **p < 0.01 by t test.

Oil Red O staining of similarly stimulated myotubes at 8 d, demonstrated IMCL accumulation under resistin stimulation, with an increase in Oil Red O area (37 ± 3.5 vs 26 ± 3.1 mm^2^, p < 0.05) and Oil Red O particle size (8.8 ± 0.5 vs 6.5 ± 0.2 µm^2^, p < 0.01), compared to unstimulated controls (Fig. 6A,B). A Seahorse XF Palmitate-BSA FAO Substrate Mitochondrial Stress Test, carried out on a Seahorse XFe96 extracellular flux analyser demonstrated that resistin, compared to unstimulated control, significantly increased exogenous (0.15 ± 0.03 vs 0.02 ± 0.01 pmoles/min/µg, p < 0.01) fatty acid oxidation by myotubes (Fig. 6C) and promoted endogenous fatty acid oxidation, a phenomenon that was not detectable in unstimulated cultures (Fig. 6D).

## Discussion

In this study, we describe for the first time the adverse myogenic effects of the obese human SAT secretome, generated using conditioned media. The stromal vascular fraction of adipose tissue is a more prolific secretor of pro-inflammatory cytokines than mature adipocytes^25^ and thus our experimental model may be a more physiologically relevant model of adipose tissue adipokine secretion than models which rely upon adipokine secretion by adipocytes alone^24^. Furthermore, since SAT represents a much larger proportion of total adipose tissue mass than VAT^27–29^, its contribution to the systemic inflammatory burden to which skeletal muscle is exposed is likely to be significant. Indeed, myotubes from elderly subjects cultured with obese SAT secretome were 30% thinner and had a 40% reduction in the number of nuclei incorporated into myotubes, compared to those cultured with normal weight SAT conditioned media. Previous work by Pellegrinelli *et al*. demonstrated that the lean subcutaneous adipocyte inflammatory secretome does not have a detrimental effect on myotube formation in neonatal myogenic cultures, but that the obese VAT adipocyte secretome does inhibit myotube formation in such cultures^24^. Interestingly, adipocytes isolated from obese SAT showed an intermediary negative effect on MTT between lean SAT and obese VAT^24^. Our work builds on these observations by the inclusion of both lean and obese SAT inflammatory milieu, using whole adipose tissue rather than adipocytes to generate conditioned medium and by demonstrating an anti-myogenic effect of OB ACM on elderly (but not young) adult human myogenic cultures. Our results suggest that younger skeletal muscle may be intrinsically more resilient to inflammatory cytokines secreted from SAT.

In considering potential secretory factors that could be - at least in part - responsible for the effect of the obese SAT secretome on myogenesis, the concentration of resistin was found to be significantly elevated in the SAT conditioned media secretome collected from non-lean (BMI > 25) older individuals, compared to that collected from lean (BMI < 25) older individuals. Resistin is a pro-inflammatory adipokine that is produced predominantly by monocytes and macrophages in humans, with a smaller proportion being produced by adipocytes^43^. Given the importance of adipose tissue M1 macrophage accumulation in ageing and obesity^44–47^, adipose tissue secretion of resistin may be of significant consequence in sarcopenia. However, few studies have previously described the effect of resistin on human skeletal muscle and sarcopenia. Plasma resistin concentrations have been reported to have an inverse relationship with quadriceps torque in old (69–81 yr), but not in young (18–30 yr), subjects^11^. A recent study has described an inverse relationship between abdominal skeletal muscle density and systemic resistin concentrations^48^; such increases in skeletal muscle density are thought to indicate improved muscle quality and have been associated with increased muscle strength^49^. Furthermore, C2C12 mouse myoblast proliferation is increased by the transfection of a human resistin eukaryotic expression vector, and such transfection reduces the expression of desmin and results in thinner myotubes^50^. We thus identified resistin as warranting further exploration of its myogenic effects.

Here, we demonstrate that stimulation of developing myotubes with recombinant resistin, at a concentration reported physiologically in older humans^30^, has a substantial detrimental effect on the formation of such myotubes. Notably, myogenic cultures from both young and old subjects were thinner following resistin stimulation, but only old myotubes displayed a reduction in their NFI. Similarly, obese SAT conditioned media that contained more resistin had a greater detrimental effect on both myotube thickness and NFI of elderly myotubes compared to young myotubes. This might indicate that there are age-related differences in the *ex vivo* myogenic capacity of myoblasts under inflammatory conditions, with young muscle being more resistant to pathological levels of adipokines such as resistin than older muscle. The mechanisms underlying the differential responses of young and elderly myotubes to OB ACM were not explored in this study, yet plausible avenues of enquiry exist. Primary human myogenic cultures are known to retain some of the characteristics of their donors^51–53^. Furthermore, aged skeletal muscle displays increased classical NFκB pathway activity^54,55^. It is possible altered cytokine receptor expression levels leave elderly myogenic cultures more susceptible to the detrimental effects of OB ACM on culture differentiation. However, we are unaware of any comprehensive profile of cytokine receptor gene or protein expression comparing young and old human skeletal muscle.

Given these findings, it is highly significant that depletion of resistin from obese SAT secretome reduced the anti-myogenic action of obese SAT conditioned media secretome. However, it is important to note that there are likely to be additional factors within obese SAT conditioned media, which we did not assess, that could have contributed to the considerable declines in both myotube thickness and NFI we observed in elderly myotube cultures. Indeed, the recent paper by Saini *et al*.^23^, highlighted that the combination of pro-inflammatory cytokines and saturated fats can exacerbate anti-myogenic activity, suggesting that *in vivo* it is likely that it is the complex interactions between multiple factors within the adipose tissue inflammatory milieu that mediate muscle decline.

It is clear from the literature that resistin activates NFκB signaling. Such activation has been demonstrated in the HepG2 cells^36^, human coronary artery endothelial cells^38^ and in human macrophages^37^. Importantly, genetic approaches have now established the classical NFκB pathway as a negative regulator of myogenesis. Myogenesis has been shown to be enhanced in p65^−/−^ myoblasts^34^, whilst the IKKβ inhibitor IV has been shown to enhance the myogenic differentiation of primary murine cultures from wild-type mice^35^. Furthermore, NFκB activation in the satellite cells of aged mice inhibited skeletal muscle regeneration in response to cryoinjury^56^.

In myogenic cultures, classical NFκB pathway activity is diminished at 48 h post-differentiation^34^. Importantly, we observed that the addition of recombinant resistin to our myogenic cultures resulted in persistent p65 phosphorylation (indicative of NFκB activation) at 48 h, a phenomenon that was reversed by the addition of the IKK2 inhibitor TPCA-1. Importantly, TPCA-1 rescued the differentiation of our myogenic cultures in the presence of recombinant resistin, suggesting therefore that resistin impaired myogenesis *via* activation of the classical NFκB pathway.

We further observed that resistin promoted the accumulation of IMCL in human myotube cultures, as well as promoted an increased reliance on fatty acid oxidation to sustain an overall increase in metabolic activity, which may reflect the presence of cellular metabolic stress. None the less, the increase in myotube metabolic activity in the presence of diminished myotube formation is surprising. Since we observed no difference in mitochondria number between resistin stimulated and unstimulated myotubes, the increase in respiration could be indicative of metabolic stress. Indeed, futile substrate cycling between *de novo* lipogenesis and fatty acid oxidation had been previously reported in *ex vivo* murine skeletal muscle upon stimulation with the adipokine leptin^57^, and might partly explain our observations. It must be noted however that *de novo* lipogenesis is thought to be limited under normal physiological conditions in skeletal muscle^58^. The observation of increased fatty acid oxidation and IMCL accumulation is in agreement with reports of lipid accumulation in resistin-stimulated human macrophages^59^ and of hepatic steatosis in resistin-treated mice^36^. Therefore, our observation of enhanced exogenous fatty acid oxidation and accumulation of IMCL in human myotubes under resistin stimulation conditions, coupled with observations by others that resistin stimulation of human macrophages promotes IMCL accumulation and the cell surface expression of the fatty acid transporter CD36^59^, suggests that enhanced fatty acid uptake – in excess of its increased oxidation by human myotubes – is responsible for the IMCL accumulation described in the present study. Dysfunctional intramyocellular fatty acid handling and IMCL accumulation are associated with both skeletal muscle insulin resistance^60,61^, and anabolic resistance^62^. Furthermore, an increase in lipid content in type I fibres appears to be a feature of sedentary, but not physically active ageing^63^. The effect of IMCL accumulation on skeletal myogenesis is less well established, but O-GlcNAcylation, a post-translational modification that is associated with obesity and diabetes, has a detrimental effect on C2C12 myogenesis^40^. Given these findings, the functional effects of resistin on muscle metabolism we report here may have important implications. However, further studies are required to elucidate the mechanism by which resistin impacts on muscle metabolism by investigating the precise nature of the relationship between the myogenic and metabolic responses to resistin stimulation in human muscle cells and their importance for substrate regulation and insulin action.

This study has some limitations. Firstly, our young and elderly participants were recruited for biopsies at different times. Therefore, it was not possible to directly compare the differences between elderly and young baseline NFI and MTT. Whilst we made every effort to standardise culture conditions, differences in media serum batches could have impacted on the myogenic differentiation. Thus, in this study we have restricted our statistical analysis of the data to comparing datasets to their own controls (unstimulated vs resistin). Secondly, we sourced commercially available myoblasts to complete the validation of resistin on myogenesis in the absence of access to further skeletal muscle biopsies. These commercial cells were isolated from a young person and were validated as sensitive to the anti-myogenic effects of resistin. In summary, our studies describe a detrimental effect of obese SAT conditioned media secretome on primary human myogenesis and identify resistin as the adipokine that - at least in part - mediates this effect. Furthermore, we demonstrate that resistin exerts its anti-myogenic effects by causing persistent activation of the classical NFκB pathway, and alterations in muscle metabolism. These findings may have important implications for the maintenance of muscle mass and metabolic control in older people who are obese or overweight, or those with chronic conditions such as osteoarthritis^30^ and type 2 diabetes^64^, which are associated with increased levels of resistin in the circulation.

## Electronic supplementary material

Dataset 1
