# Supplementary material for: Obese subcutaneous adipose tissue impairs human myogenesis, particularly in old skeletal muscle, via resistin-mediated activation of NFκB
Source: Sci Rep. 2018 Oct 18;8:15360. doi: 10.1038/s41598-018-33840-x (PMC6193975; doi:10.1038/s41598-018-33840-x)
Supplement: Supplementary file 1 — Dataset 1 [file 41598_2018_33840_MOESM1_ESM.docx]

**Supplementary Data**

**Title: Obese subcutaneous adipose tissue impairs human myogenesis, particularly in old skeletal muscle, via resistin-mediated activation of NFκB**

Mary F. O’Leary, Graham R. Wallace, Edward T Davis, David P Murphy, Thomas Nicholson, Andrew J. Bennett, Kostas Tsintzas, Simon W. Jones

Supplementary Figure 1

Supplementary Figure 2

Supplementary Figure 3

Supplementary Figure 4

Supplementary Figure 5

Supplementary Figure 6

Supplementary Figure 7


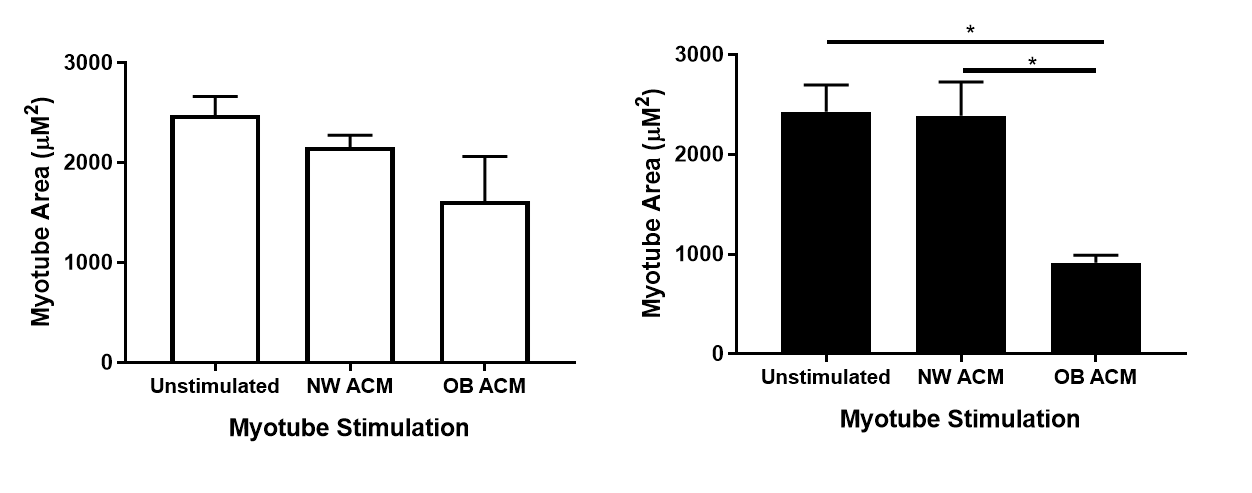


**Supplementary Figure 1. The effect of obese and normal-weight adipose conditioned media secretome on myotube area in young and old subjects.** Subconfluent myoblasts from young (18-30 yr old; white bars)) and old (>65 y old, black bars) were switched to unconditioned differentiation medium or differentiation medium that had previously been conditioned with adipose tissue from normal weight (NW ACM) or obese individuals (OB ACM, n = 3; BMI>30 kg/m^2^ ). Media were renewed every 2 d. At 8 d, myotubes were fixed, immunofluorescence stained for desmin and with DAPI and imaged on an epifluorescence microscope. Data represents the mean ± SEM of n=3 biological replicates.


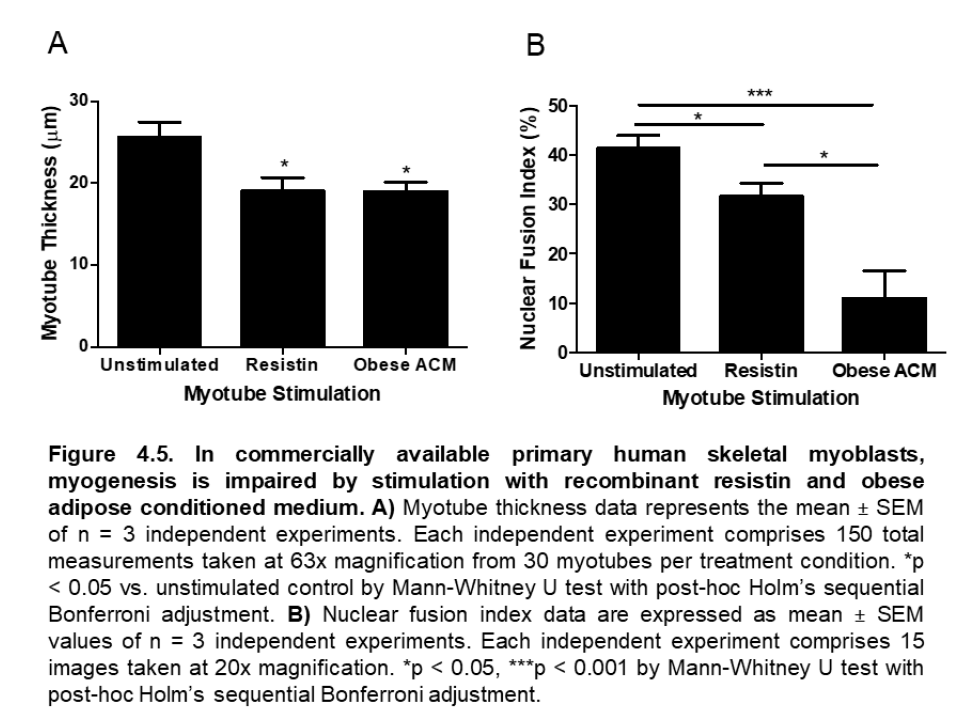


**Supplementary Figure 2. Resistin and obese ACM impair myogenesis in commercially available primary human skeletal myoblasts**. (**A**) Myotube thickness data represents the mean ± SEM of n=3 independent experiments. Each independent experiment comprises 150 total measurements taken at 63x magnification from 30 myotubes per treatment condition. *p<0.05 vs unstimulated control. (**B**) Nuclear fusion index data are ex mean ± SEM of n=3 independent experiments. Each independent experiment comprises 15 measurements taken at 20x magnification. *p<0.05, ***p<0.001.


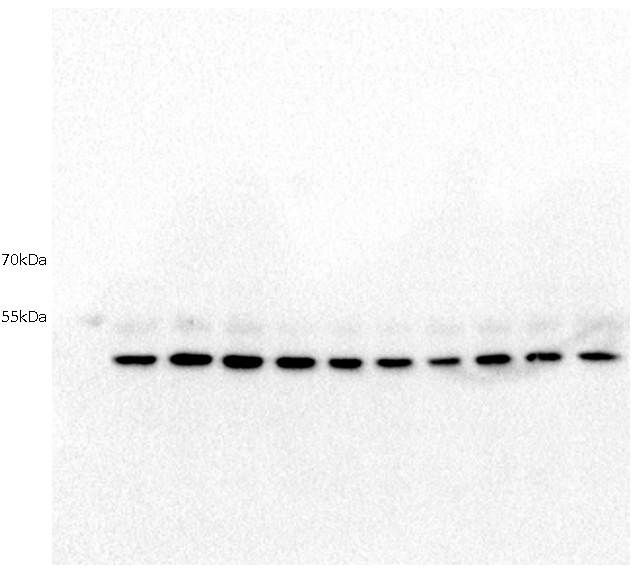

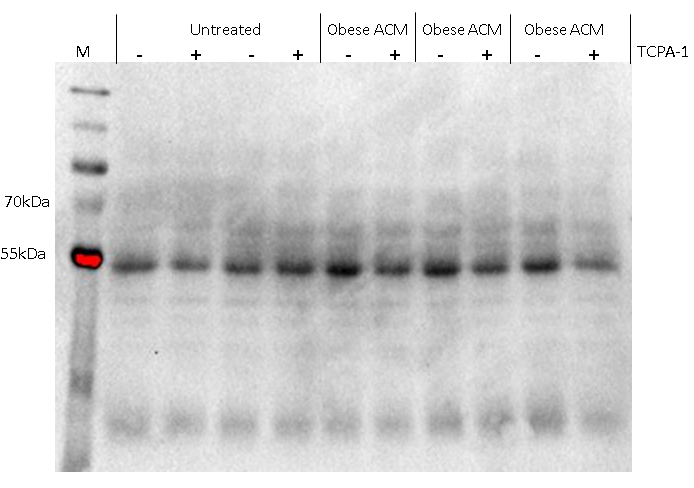


**B**

**A**

**Supplementary Figure 3. Obese ACM activates NFkB.** Subconfluent primary human skeletal myoblasts were switched to either normal differentiation growth media (untreated) or to differentiation media containing obese ACM from n=3 different patients with or without 40 nM TPCA-1 for 48h. Protein lysates were quantified for total protein concentration by BCA assay and aliquots of equal total protein load were analysed for the expression of phospho-p65 by Western blotting. (**A**) Immunoblot of phospho-p65 (Ser536) and beta-actin. M represents the molecular weight marker lane. (**B**) Bars represent mean densitometric units of phospho-p65/beta-actin ± SEM values. * = p<0.05


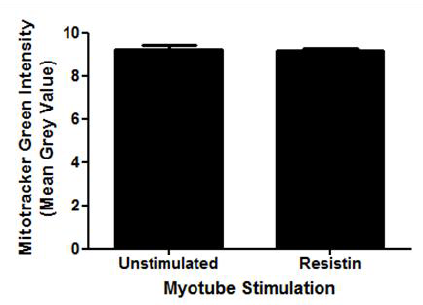


**Supplementary Figure 4. The effect of resistin on myotube mitochondrial number.** Myotubes were differentiated with or without recombinant resistin (5ng/ml) for 8 days and then stained with 100nM Mitotracker Green and imaged on a fluorescent microscope. Fluorescent intensity was quantified using Image J.


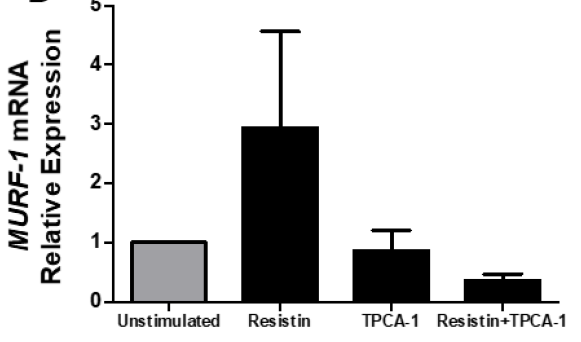

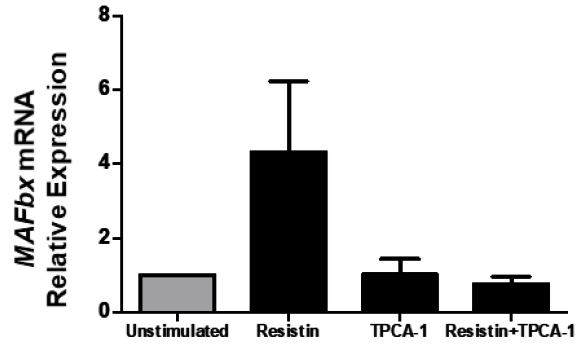


**Supplementary Figure 5. The effect of resistin on the expression of the muscle-specific E3 ligases MAFbx and MURF-1.** Myotubes were differentiated for 8 days with or without recombinant resistin (5ng/ml). Total RNA was extracted and the expression of MAFbx and MURF-1 was determined by qRTPCR. Data represents the mean ± SEM of 3 independent experiments.


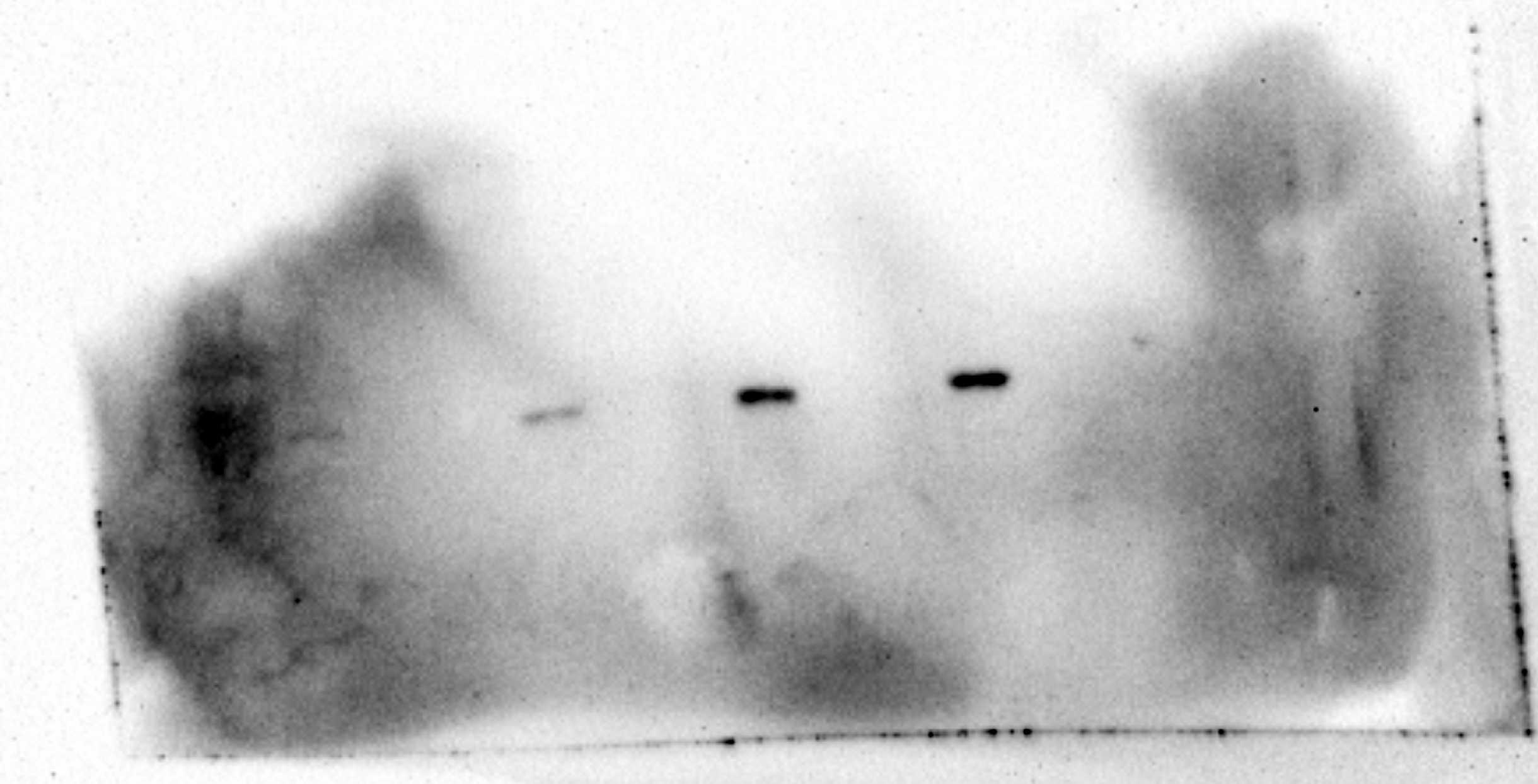


**Supplementary Figure 6**. Full size of immunoblot of resistin. Predicted MW - 12.5 kDa

20kDa


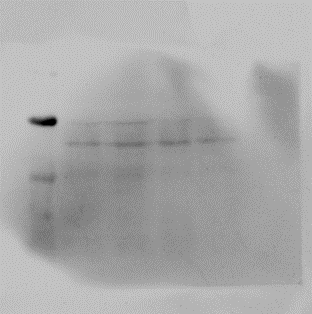


70kDa

50kDa

35kDa

pp65 (Ser 536)

Predicted – 60 kDa


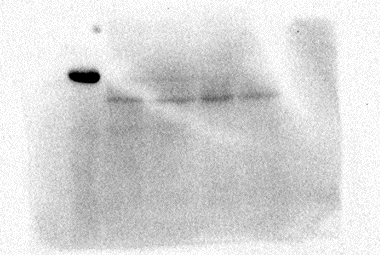


70kDa

50kDa

35kDa

p65

Predicted – 60 kDa

**Supplementary Figure 7**. Full size of immunoblot of phospho and total p65
